# Proteomic Identification and Functional Analysis of *Babesia microti* Reveals Heparin-Binding Proteins

**DOI:** 10.1155/jotm/8821002

**Published:** 2025-01-11

**Authors:** Yu Chun Cai, Bin Xu, Yan Hong Chu, Ying Fang Yu, Jia Hui Sun, Zi Ran Mo, Han Yin Yang, Shu Ning Yan, Mu Xin Chen, Jia Xu Chen

**Affiliations:** ^1^National Institute of Parasitic Diseases, Chinese Center for Disease Control and Prevention (Chinese Center for Tropical Diseases Research), Laboratory of Parasite and Vector Biology, Ministry of Public Health, WHO Collaborating Centre for Tropical Diseases, National Center for International Research on Tropical Diseases, Ministry of Science and Technology, Shanghai 200025, China; ^2^National Key Laboratory of Intelligent Tracking and Forecasting for Infectious Diseases, Chinese Center for Disease Control and Prevention (Chinese Center for Tropical Diseases Research), National Institute of Parasitic Diseases, Shanghai 200025, China; ^3^The Institutes of Biomedical Sciences, College of Life Sciences, Inner Mongolia University, Hohhot 010000, China

**Keywords:** *Babesia microti*, Babesiosis, heparin-binding protein, invasion of host, protein-protein interaction

## Abstract

Glycosaminoglycan (GAG) molecules on the surface of red blood cells play an important regulatory role in the invasion of merozoites of apicomplexan protozoa. Heparan sulfate, a type of GAG molecule, has been identified as an important receptor facilitating the invasion of red blood cells by these parasites. Proteins in the parasite that exhibit strong affinity for heparin may play a pivotal role in this invasion process. This study aims to use proteomics to identify *Babesia microti* proteins with high binding affinity to heparin. Bioinformatics was utilized to analyze the subcellular localization and biological functions of these proteins. Candidate genes encoding proteins with strong heparin affinity will be expressed in a prokaryotic system to produce recombinant proteins. The interaction between these recombinant proteins and heparin will be characterized through heparin-binding experiments and other methods. Initially, a mouse model of *B. microti* was established and high-density *B. microti* were obtained. Heparin affinity chromatography was then used to purify natural *B. microti* proteins that can bind to heparin, identifying 186 *B. microti* proteins via ESI-MS that specifically interact with heparin. Further studies were carried out to analyze those specific proteins with unique peptide segments of two or more, yielding 15 *B. microti* proteins, most of which are cell surface proteins and secretory proteins. Based on mass spectrometry identification and subsequent analyses, BMSA5-1-1, *B. microti* peptidyl-prolyl cis-trans isomerase (BmPPIase), and chaperonin were selected for further study due to their potential impact on the invasion of red blood cells by *B. microti*. These candidate proteins were expressed as recombinant proteins using a prokaryotic expression system. In vitro heparin-binding assays demonstrated that these recombinant proteins specifically bind to heparin. Notably, BmPPIase and chaperonin recombinant proteins exhibited activity in specific heparin binding. Molecular interaction studies further confirmed the strong interaction between BmPPIase and heparin. In conclusion, this study used proteomic methods to identify 186 specific *B. microti* proteins with specific binding affinity to heparin, providing in-depth analysis of 15 key proteins. The findings confirmed that BmPPIase and chaperonin specifically bind to heparin, with molecular interaction experiments substantiating the strong interaction between BmPPIase and heparin.

## 1. Introduction

Babesiosis is a zoonotic blood-borne protozoan disease in which *Babesia* invades the host's red blood cells, typically transmitted through tick bites, blood transfusions, or blood products [1]. In recent years, Babesiosis has been designated as a legally notifiable infectious disease in many countries, emerging as a significant global health threat. In China, Babesiosis is a newly emerging parasitic disease, with reported cases across multiple regions [2]. Among the reported cases in China, one was documented in Zhejiang Province [3], two cases in Taiwan [4, 5], ten in Yunnan Province [6], and one in Guangxi Province—all confirmed to be caused by *Babesia microti*. Heilongjiang Province reported 48 confirmed cases of *B. venetorum* infection, along with 16 suspected cases [7, 8]. Additionally, in 2016, a novel *Babesia* species infection was reported in Shanghai [9]. The number of reported cases of *Babesia* infection has been steadily increasing each year. Due to Babesiosis being a newly emerging parasitic disease, there is a significant gap in knowledge, skills, and technical resources concerning the pathogen's biology, clinical diagnosis, treatment, and strategies for prevention and control. There is an urgent need for scientific research on this disease in China.

Merozoites of *Babesia* develop and reproduce within red blood cells. The extensive proliferation of *Babesia* can cause red blood cells to rupture, leading to severe hemolysis in the host. This rupture release a new wave of merozoites that continue to infect additional red blood cells, resulting in clinical symptoms such as high fever, anemia, jaundice, and hemoglobinuria in the host [10]. Therefore, understanding the process of merozoite invasion into red blood cells and identifying key proteins involved in the interaction between *B. microti* merozoites and host red blood cells is essential for the diagnosis and prevention of *Babesiosis*.

Heparan sulfate (HS), blood group glycoproteins A, B, C, glucose converting protein, and anion transport proteins are key proteins on the red blood cell membrane. With increasing research into the invasion mechanisms of protozoa such as *Toxoplasma gondii*, *Plasmodium falciparum*, *Babesia*, and other protozoa, growing evidence suggests that HS on the surface of red blood cells serves as an important receptor for these invasions. Glycosaminoglycan (GAG) molecules play an important regulatory role in the invasion of protozoa into host red blood cells by merozoites [11, 12].

HS is a widely distributed GAG, found in all tissue cells, and is composed of polysaccharides and protein molecules. It is a protein polysaccharide molecule, consisting of core proteins connected to linear sugar chains [10, 13, 14]. Negatively charged GAG molecules such as HS can bind to specific amino acid sequences, mediating the initial adhesion between red blood cells and the parasite. Chen et al. [10, 14] first confirmed the mechanism by which heparin treats malaria, demonstrating that the receptor adhered to the surface of red blood cells infected by *P. falciparum* is heparin disulfide.

Proteins such as variant merozoite surface antigens (SAGs) (VMSAs) [15], apical membrane antigens (AMAs) [16], and thrombomodulin-related adhesion proteins (TRAP) [17] have been identified as key factors in the invasion of red blood cells by *Babesia* species such as *B. bovis* and *B. gibsoni*. However, the specific receptors and adhesion mechanisms involved in their interactions with host cells remain unclear. Research on *Babesia* in field mice is even more limited, and the existence of homologous proteins and their interaction mechanisms with red blood cells in *B. microti* still require systematic investigation. HS, a cell receptor found on the surface of all tissue cells, has showed to play an important role as a red blood cell surface receptor in the invasion of parasites such as *Plasmodium* and *T. gondii*, mediating the initial adhesion between red blood cells and parasites. This study aims to obtain protein information through reverse proteomics identification and conduct biological analysis on recombinant proteins, with the goal of identifying proteins related to *Babesia* and their role in the invasion of red blood cells in field mice through functional analysis.

## 2. Materials and Methods

### 2.1. *B. microti* Strain and Experimental Animals

The *B. microti* strain used in this study was the Peabody mjr (PRA-99) strain, obtained from the American Type Culture Collection (ATCC). The PRA-99 strain of *B. microti* was under long-term conservation. The experimental mice were purchased from the Shanghai Experimental Animal Center of the Chinese Academy of Sciences (animal license number: SCXK [Shanghai] 2012-002). The infected mice used in this study were BALB/c mice, infected with the PRA-99 strain, and were used for long-term animal conservation. All animal experiments conducted in this study were approved by the Experimental Animal Welfare and Ethics Committee of the Animal Center of the Institute of Parasitic Disease Prevention and Control and the Chinese Center for Disease Control and Prevention (Chinese Center for Tropical Diseases Research) (Animal Ethics Approval No: IPD-2021-4).

### 2.2. Establishment and Maintenance of the Animal Model for *B. microti*

After retrieval from liquid nitrogen, the *B. microti*–spiked blood was allowed to thaw and settle at room temperature before being inoculated intraperitoneally into mice at a dose of 100 *μ*L per mouse. The proliferation of *B. microti* was monitored by collecting tail blood, preparing thin blood smears, and staining them with Giemsa for microscopic examination. During evaluation, 1000 red blood cells were counted per slide to determine the number of infected cells and calculate the infection rate. The infection rate was determined as ([number of infected red blood cells/1000] × 100). The average rate from three slides per group was recorded as the daily infection rate for that group. Once the peak infection rate in the BALB/c mice's red blood cells reached approximately 70% to 80%, the infected blood was diluted and used to inoculate healthy BALB/c mice for subsequent passages.

### 2.3. Extraction of *B*. *microti* Proteins

Whole blood was collected from BALB/c mice infected with *B. microti* at the peak (about 70%∼80%). Red blood cells and plasma were isolated from the blood. Briefly, 6 mL of 40% Percoll and 3 mL of 70% Percoll were added to the blood cells, followed by centrifugation. The lower layer of red blood cells was collected and treated with five volumes of red blood cell lysis buffer, then incubated on ice for 15 min. The mixture was centrifuged at 450 g for 10 min, and the supernatant was transferred to a 1.5 mL centrifuge tube and centrifuged at 12000 g for 10 min. The resulting pellet, containing *B. microti*, was collected and resuspended in PBS buffer. The suspension was centrifuged at 800 g for 10 min, washed three times, and a protease inhibitor (phenylmethanesulfonyl fluoride, PMSF) was added to a final concentration of 1 mmol/L. The suspension was then subjected to five freeze-thaw cycles at −80°C, sonicated, and centrifuged at high speed (13,000 g) for 1 h. The supernatant, containing the *B. microti* protein, was collected, and the protein concentration was measured. The protein extract was stored at −20°C for future use.

### 2.4. Experiment on the Binding of *B*. *microti* Protein and Heparin

*B.microti* protein at a concentration of 1 mg/mL was incubated with equal volumes of heparin Sepharose or Sepharose at 4°C for 2 h, respectively. The mixture was then washed three times with PBST buffer and eluted using a 2-M NaCl solution. Proteins that bound to heparin Sepharose or Sepharose were subjected to 12% SDS-PAGE electrophoresis, respectively. The electrophoresis results were analyzed using the Multi Genius Bio imaging system (Syngene, England).

### 2.5. Collection of Heparin-Binding Proteins From *B. microti*

The purification and collection of heparin-binding proteins were performed using an AKTA protein purifier and Unicorn software. The purification process began by connecting the HiTrap heparin column to the protein purifier and washing both the column and the entire purification system with 10 column volume of binding buffer at a flow rate of 0.1 mL/min. The total of *B. microti* protein extract was dissolved in the binding buffer and filtered through a 0.45 μm filter. The filtered solution was loaded onto the column using a 1 mL syringe. After loading, the column was washed with 10 column volumes of elution buffer, and the fractions were collected. The eluate was concentrated and dialyzed, and the dialyzed protein was stored at −80°C for future use.

### 2.6. Identification of Heparin-Binding Proteins From *B. microti* by ESI-MS/MS Method

ESI-MS/MS method was used to identify heparin-binding proteins from *B. microti*. The process involved enzymatic hydrolysis of proteins in the gel using trypsin for 20 h, followed by extraction of the hydrolyzed peptide segments; these segments were then analyzed using ESI mass spectrometry to obtain mass spectrometry data. Detailed mass spectrometry identification and search parameters are provided in the Supporting Information of Supporting Table 1. The Bioworks browser 3.3 software was used to retrieve the relevant database and analyze the identified proteins.

### 2.7. Expression and Purification of Recombinant Proteins of *B. microti*

This study employed whole-gene synthesis to produce the target gene. The target gene was double-digested with BamHI and XhoI restriction endonucleases according to the specified reaction conditions. The pET28a vector, which had been successfully ligated with the target gene, was transformed into *E. coli* DH5*α* competent cells. The recombinant plasmid was then transferred into *E. coli* BL21 (DE3) competent cells and cultured in LB medium at 200 rpm and 37°C for 1 h. Afterward, the culture was centrifuged at 3000 rpm for 10 min to collect the bacterial pellet. The resuspended cells were plated onto LB agar plates containing kanamycin and incubated overnight at 37°C. A 1% aliquot of the overnight culture was grown in kanamycin-resistant medium for 3 h and then induced with 0.5 mM IPTG at 30°C for 4 h. Subsequently, the *E. coli* cultures were harvested and sonicated, and both the pellet and supernatant were collected. The solubility of the recombinant proteins was assessed via SDS-PAGE. *E. coli* BL21 (DE3) cells, confirmed for recombinant protein expression capability, were cultured overnight. The collected bacterial solution was sonicated, and the supernatant was collected following centrifugation. The samples were then purified using an NTA column with varying concentrations of imidazole, and the fractions were analyzed by SDS-PAGE electrophoresis.

### 2.8. Binding Experiment Between Recombinant Proteins and Heparin

The recombinant proteins were incubated with equal volume of heparin Sepharose and Sepharose 4B for 2 h. The mixture of recombinant protein with heparin Sepharose and recombinant protein with Sepharose 4B were washed three times with 10 column volumes of PBST buffer. After washing, the proteins bound to heparin Sepharose and Sepharose 4B were eluted with a 2 M NaCl solution. The eluted samples were then analyzed by 12% SDS-PAGE and Western blotting.

### 2.9. Recombinant Protein–Heparin Interaction Experiment

This study employed a molecular interaction analyzer to investigate the interaction between recombinant proteins and heparin, determining their binding and dissociation constants. The recombinant protein, serving as the ligand, was diluted in acetic acid solutions with pH values of 4.0, 4.5, 5.0, and 5.5 to a concentration of 2 μg/mL for pH scouting tests. These tests led to the selection of a pH 5.5 acetic acid solution at a concentration of 10 μg/mL as the optimal buffer for immobilizing the ligand protein on the chip. The immobilization process involved chip activation for 7–10 min, ligand coupling for 400 s, and chip closure for 7 min to complete ligand fixation. HS was prepared at an initial concentration of 2000 *μ*M, and then diluted in a gradient to 12 concentration points for multicycle kinetic operation. The parameters for the analysis included binding in PBS-Tween-20 (0.05%) buffer for 120 s and dissociation for 240 s. The raw data were imported into BiacoreTM Insight Evaluation Software 4.0.12.15655, where a multicycle kinetic analysis was performed. At least five concentration points were selected, and a 1:1 binding fitting model was used to fit the kinetic curves, resulting in the calculation of the binding rate constant (Ka), dissociation constant (Kd), and affinity constant (KD).

## 3. Results

### 3.1. Enrichment of *B. microti* and Extraction of Natural Proteins From *B. microti*

This study utilized Percoll gradient centrifugation to purify *B. microti* from a mice model. A 40% Percoll solution was added to the centrifuged blood cells, followed by the slow addition of a 70% Percoll solution from the bottom of the tube, after which the mixture was centrifuged. Following centrifugation, white blood cells and other nonred blood cells were found suspended between the 40% and 70% Percoll layers, while all infected and uninfected red blood cells settled below the 70% Percoll layer. The enriched sample was stained with Giemsa solution, revealing the characteristic blue dot-shaped *B. microti* under the microscope (Figure 1 in the Supporting information). The enriched *B. microti* was then subjected to freeze-thaw cycles and ultrasonic fragmentation, and the supernatant was collected to obtain soluble *B. microti* proteins. Analysis of these proteins by SDS-PAGE revealed five major bands with molecular weights of 72, 55, 43, and 17 kDa, along with seven secondary bands ranging from 14.4 to 95 kDa (see Figure 2 in the Supporting Information).

### 3.2. Heparin Binding and Affinity Purification of Natural Proteins From *B. microti*

**Figure 1:**
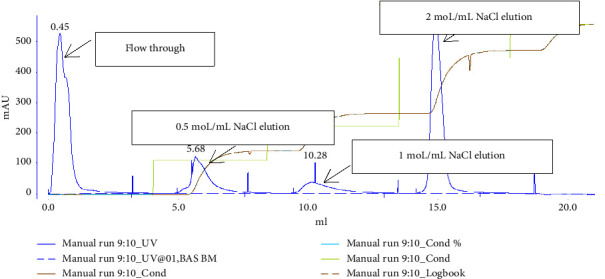
Purification of heparin-binding protein of *B. microti*. The initial flow-through was collected, producing a peak detected by the system. The samples were eluted using 0.5, 1, and 2 M sodium chloride elution buffers. No peaks were observed with the 0.5- and 1 M sodium chloride solutions. A peak appeared with the 2 M sodium chloride solution, indicating the elution of proteins that were tightly bound to heparin.

The native proteins of *B. microti* were loaded into a protein purifier and washed with a loading buffer. The initial flow-through was collected, producing a peak detected by the system. Subsequently, the samples were eluted using 0.5, 1, and 2 sodium chloride elution buffers. No peaks were observed with the 0.5- and 1-M sodium chloride solutions; however, a peak appeared with the 2-M sodium chloride solution, indicating the elution of proteins that were tightly bound to heparin (see Figure 1). Based on these results, the native *B. microti* proteins were combined with pretreated heparin Sepharose and Sepharose, followed by elution with 2 M sodium chloride. The sample was analyzed by SDS-PAGE electrophoresis before and after treatment (see Figure 3 in the Supporting Information). It was found that the *B. microti* proteins bound to heparin Sepharose, while they did not bind to regular Sepharose.

### 3.3. Mass Spectrometry Data Identification and Analysis

**Figure 2:**
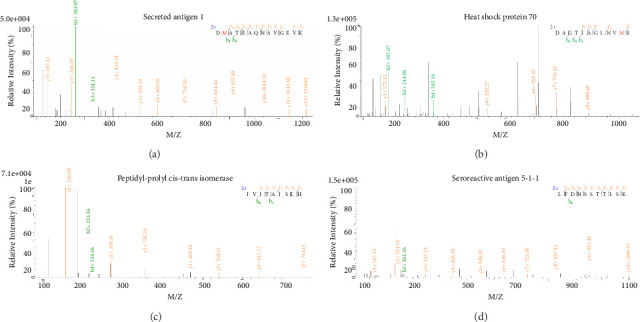
Secondary mass spectrometry of the important heparin-binding proteins (a) for secondary mass spectrometry of BMSA1, (b) for secondary mass spectrometry of HSP70, (c) for secondary mass spectrometry of BmPPIase, and (d) for secondary mass spectrometry of BMSA5-1-1.

To identify the native proteins of *B. microti* that bind to heparin Sepharose, we performed ESI-MS mass spectrometry on the proteins eluted with 2 M sodium chloride elution buffer. The mass spectrometry analysis lasted 60 min, with an NL index of 2.61E9, meeting the necessary criteria. Using BioWorks browser 3.3 software, we searched and matched the reference sequences from *Babesia*'s protein database against the raw data. This analysis identified 186 proteins from 123 protein groups, of which 26 proteins had two or more unique peptide segments, accounting for 21% of the total protein count. Among these, 21 proteins were from *B. microti*, two proteins were from *B. equi*, and 1 protein each was from *B. bigemina*, *B. bovis*, and *B. vitalii*. The four proteins with the highest number of unique peptide segments were Secret antigen 1 (fragment), Heat shock protein (HSP) 70, BmPPIase, and Seroactive antigen 5-1-1, respectively (see Figure 2).

In the identified heparin-binding proteome of *B. microti,* there were 15 proteins with more than three unique peptide segments, all originating from *B. microti* PRA-99 or *B. microti* strain RI (see Table 1). These included seven surface and secreted antigen proteins, such as secret antigen 1, SAG, seroactive antigen 5-1-1, N1-21 subtype b protein, N1-21 subtype a protein, and seroactive antigen BMN1-3. Additionally, there were HSP family proteins such as HSP 70 (P90655), HSP70 (G0ZI00), and chaperonin protein (I7IPV2), as well as proteases such as BmPPIase.

### 3.4. Expression and Purification of Recombinant Heparin-Binding Protein of *B. microti*

Bioinformatic analysis was conducted on 15 important heparin-binding proteins from *B. microti*. Based on expression difficulty, three proteins including chaperonin, BmPPIase, and Seroactive antigen 5-1-1 were selected for recombinant expression and further functional analysis. The BmPPIase gene was cloned into the pET28a vector (pET28a-BmPPIase) and expressed in *E. coli* BL21 (DE3), producing a recombinant BmPPIase protein with an approximate molecular weight of 19 kDa. Additionally, a 378-bp fragment of BmSA5-1-1 was inserted into the pET28a vector using BamHI and XhoI restriction enzymes. The inserted gene was expressed and purified in *E. coli* BL21 (DE3), yielding a recombinant BmSA5-1-1 protein of 15 kDa. Indirect immunofluorescence (IFA) revealed that BmSA5-1-1 is a secretory protein localized on the surface of *B. microti*. The sequences, cloning, and expression processes of BmPPIase and BmSA5-1-1 are detailed in the Supporting Information of Supporting Figures 4 and 5 [18, 19].

**Figure 3:**
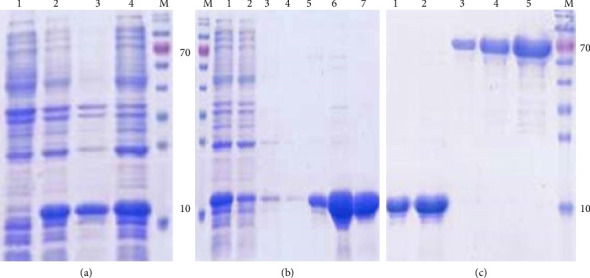
Expression and purification of chaperonin protein: (a) M represents protein marker, Lane a1 represents preinduced protein, Lane a2 represents induced protein, Lane a3 represents precipitation of induced protein, and Lane a4 represents supernatant of induced protein. (b) M represents marker, Lane b1 represents prepurified chaperonin protein, Lane b2 represents flow in liquid of chaperonin protein, and Lanes b 3-7 represent purified chaperonin protein in different concentration. (c) M represents protein marker, Lane c1 represents 1 μl recombinant chaperonin protein, Lane c2 represents 2 μl recombinant chaperonin protein, Lane c3 represents 1 μg BSA standard protein, Lane c4 represents 2 μg BSA standard protein, and Lane c5 represents 4 μg BSA standard protein.

The chaperonin gene, consisting of 339 bp, was cloned into the pET28 expression vector using Bam*HI* and Xho*I*, and the construct was transformed into *E. coli* BL21 (DE3). Induction with 0.5 mM IPTG at 30°C for 4 h resulted in the expression of recombinant chaperonin protein, which was approximately 12 kDa in size. The expression was confirmed by 15% SDS-PAGE electrophoresis on total bacterial lysates before and after induction, showing a clear, specific induction band near the expected size (Figure 3). An additional 15% SDS-PAGE electrophoresis revealed that the chaperonin protein was primarily expressed in the supernatant, indicating soluble expression. Further analysis of NTA purification, as shown in Figure 3, confirmed that the chaperonin protein was soluble, and it was eluted at imidazole concentrations between 200 and 500 mM, with a protein concentration of approximately 1.0 mg/mL.

### 3.5. Analysis of Binding Characteristics Between Recombinant Proteins and Heparin

**Figure 4:**
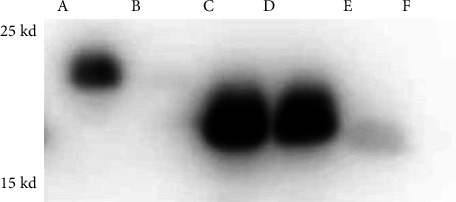
Western blot results of recombinant protein binding to heparin. Lane A represents heparin Sepharose binding to BmPPIase, Lane B represents Sepharose 4B binding to BmPPIase, Lane C represents heparin Sepharose binding to BmSA5-1-1, Lane D represents Sepharose 4B binding to BmSA5-1-1, Lane E represents heparin Sepharose binding to chaperonin, and Lane F represents Sepharose 4B binding to chaperonin.

The recombinant proteins—BmPPIase, BMSA5-1-1, and chaperone—were each combined with heparin to assess their binding ability with heparin. Western blot results demonstrated that both BmPPIase and chaperone protein bound to heparin Sepharose, as indicated by prominent bands, while neither protein bound to Sepharose 4B. This suggests that BmPPIase and chaperonin protein have strong and specific binding ability to heparin. In contrast, BmSA5-1-1 showed binding to both heparin Sepharose and Sepharose 4B, indicating a lack of binding specificity (Figure 4).

### 3.6. Analysis of the Interaction Results Between BmPPIase and Heparin Molecules

**Figure 5:**
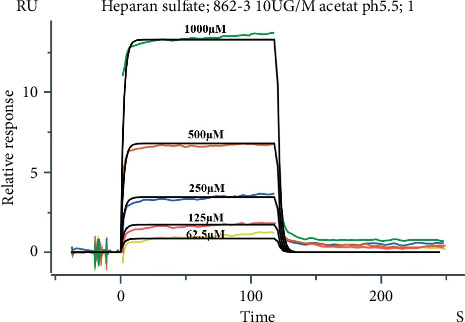
Fitting curve of the interaction between BmPPIase and heparan sulfate. Concentration gradient information, yellow represents 62.5 µM, red represents 125 µM, blue represents 250 µM, orange represents 500 µM, and green represents 1000 µM.

To further investigate the interaction between BmPPIase and heparin molecules, a 1:1 binding model of BmPPIase and heparin was used to fit the kinetic curve. The fitting results, presented in Table 2, reveal the binding rate constant (Ka) of 2.25e + 01, the dissociation constant (Kd) of 4.52e − 01, and an affinity constant (KD) of 2.01e − 02.  Figure 5 displays the fitting curves at BmPPIase concentrations of 62.5, 125, 250, 500, and 1000 μM.

## 4. Discussion

Two important members of the heparin GAG family are HS and chondroitin sulfate A (CSA), both of which play crucial roles in the infection of red blood cells by parasites [20]. Heparin GAG molecules such as fucoidan, heparin, and HS can bind to the surface of the merozoites of *T. gondii*, preventing their attachment to red blood cells and thus inhibiting parasite invasion [21, 22].

To identify proteins in *B. microti* that specifically bind to heparin, we first confirmed the presence of such proteins in *B. microti* through experiments. Using heparin agarose and agarose to interact with natural *B. microti* proteins, we found that the proteins binding to heparin agarose were eluted with high concentrations of salt, resulting in visible protein bands. However, no protein bands were observed when eluted with agarose alone, indicating that *B. microti* proteins specifically bind to heparin. This finding aligns with research on other protozoa such as *B. bovis, Plasmodium,* and *T*. *gondii*, which also possess proteins that specifically bind to heparin and play a key role in host cell invasion. In fact, heparin has showed to inhibit *B. bovis* proliferation in vitro [10]. These results suggest that *Babesia* species such as *B. microti* and *B. bovis* have the same invasion mechanism, and heparin binding proteins are essential for red blood cell invasion.

Studies have showed that various proteins secreted by different organelles of the parasite are involved in the invasion of host cells. In this study, proteins with a strong affinity for heparin were identified based on the number of unique peptide segments that bind to heparin. Among the proteins identified, 15 were found to have more than three unique peptide segments from *B*. *microti* PRA-99 or *B*. *microti* strain RI. These proteins are likely to play key roles in red blood cells invasion by *B*. *microti* (1). The most frequently identified proteins include cell surface and secretory proteins, such as secreted antigen 1 (fragment), SAG (fragment), seroactive antigen 5-1-1 (fragment), N1-21 subtype a protein, N1-21 subtype b protein, seroactive antigen BMN1-3, and six others. Notably, secret antigen 1 (BMSA1) had the highest number of unique peptide segments, supporting the idea that BMSA1 binds to heparin molecules on red blood cell surfaces, thereby influencing parasite invasion when *B*. *microti* invades red blood cells. BMSA1 is one of the most extensively studied proteins in *B*. *microti*, and diagnostic methods based on it have showed high diagnostic values. BMSA1 is the most ideal diagnostic antigen among many candidate antigens. In the study of *P. falciparum*, it has also been found that knocking out antibodies against merozoite surface protein 1 (MSP1) or the genes encoding MSP1 of *P. falciparum* reduce the infection rate, and MSP1 also works by binding to heparin molecules on the surface of red blood cells [23]. Similarly, in *T. gondii*, SAGs play a crucial role in host cell invasion by mediating the initial adhesion between *T. gondii* and host cells, followed by interactions with proteins secreted by other organelles [24]. In summary, these proteins are highly expressed on the surface of the parasite, where they mediate the initial adhesion between the parasite and host cells, and interact with heparin molecules, thereby affecting the process of parasite invasion into host cells.

The HSP family members identified in this study are HSP70 and chaperonin. HSP family proteins also play an important role in invasion. Xuan et al. obtained the recombinant protein HSP70 of *B. microti* and confirmed through in vivo and in vitro experiments that HSP70 can be a good vaccine candidate protein. Immunizing BALB/c mice with HSP70 can produce high titer-specific anti-HSP antibodies, promoting the secretion of IFN-*γ* and effectively reducing peripheral blood infection rates [25]. While this supports our hypothesis, further evidence is needed to confirm HSP70's specific binding to heparin.

To further verify the functions of the three recombinant proteins, we conducted in vitro experiments on their binding to heparin. The proteins were bound to heparin Sepharose and Sepharose and eluted with 2 M NaCl. SDS-PAGE results showed that BmPPIase and chaperone protein had strong binding affinity for heparin, indicating their potential role in the invasion process of *B. microti*. However, the BmSA5-1-1 recombinant protein bound to both heparin Sepharose and Sepharose, likely due to its insoluble nature and affected activity during purification, thus preventing its binding ability to heparin from being verified. Future studies should adjust protein expression conditions to better assess its binding ability.

The expected insertion size of the BmPPIase gene was 531 bp, and the recombinant protein of BmPPIase with a molecular weight of 19 kDa was successfully expressed. 15% SDS-PAGE electrophoresis revealed that the recombinant protein of BmPPIase was mainly expressed in the supernatant as a soluble expression [18]. Western blot analysis confirmed the strong binding of BmPPIase to heparin, laying a foundation for future studies on identifying specific binding targets. Further analysis using a protein molecular interactor revealed that BmPPIase exhibits strong interactions with heparin, suggesting its crucial role in this mechanism.

## Figures and Tables

**Table 1:** The important heparin-binding proteins of *B. microti*.

No.	Protein name	UniProt no.	Species	UniquePepCount	MW (kDa)	PI
1	Secreted antigen 1 (fragment)	E7CUK1_BABMI	*Babesia microti*	15	35.2	5.29
2	Unnamed protein	I7I937_BABMI	*Babesia microti*	15	35.4	5.29
3	Surface antigen (fragment)	J7FQS8_BABMI	*Babesia microti*	15	32.7	5.21
4	Heat shock 70 kDa protein	I7J8H8_BABMI	*Babesia microti*	10	71.6	5.02
5	Unnamed protein	I7IGN0_BABMI	*Babesia microti*	5	75.3	8.59
6	Heat shock protein 70	P90655_BABMI	*Babesia microti*	5	70.3	5.26
7	Peptidyl prolyl cis-trans isomerase	I7J5G7_BABMI	*Babesia microti*	4	21.5	9.19
8	Seroreactive antigen 5-1-1 (fragment)	B8XU05_BABMI	*Babesia microti*	3	14.8	5.8
9	Chaperonin	I7IPV2_BABMI	*Babesia microti*	3	12.1	7.95
10	Integration host factor subunit alpha	I7JDW5_BABMI	*Babesia microti*	3	18.9	9.63
11	N1-21 subtype b protein	Q869H9_BABMI	*Babesia microti*	3	34.2	5.13
12	N1-21 subtype a protein	Q869I0_BABMI	*Babesia microti*	3	34.3	5.39
13	Unnamed protein	I7JAY4	*Babesia microti*	3	32.8	5.29
14	BmGPI10, BMN1 family, N1-21a	I7IGK1_BABMI	*Babesia microti*	3	34.4	5.5
15	Seroreactive antigen BMN1-3	Q9NIP1_BABMI	*Babesia microti*	3	40.0	5.14

**Table 2:** Fitting results of the interaction between BmPPIase and heparan sulfate.

Fitting result parameters	Parameter values
Kinetics model	1:1 binding
Immobilized ligand	BmPPIase 10 UG/M acetat ph5.5
Kinetics chi^2^ (RU^2^)	1.58e − 01
ka	2.25e + 01
kd	4.52e − 01
Rmax	280.4
tc	3.79e + 11
U-value	20
Affinity constant KD (M)	2.01e − 02
Analyte 1 solution	Heparan sulfate

## Data Availability

The data that support the findings of this study are available on request from the corresponding authors. The data are not publicly available due to privacy or ethical restrictions.

## Nomenclature

HS: Heparan sulfate
VMSAs: Variant merozoite surface antigens
AMA: Apical membrane antigens
TRAP: Thrombomodulin-associated adhesion proteins
ATCC: American type culture collection
PMSF: Phenylmethanesulfonyl fluoride
Ka: Binding rate constant
Kd: Issociation constant
KD: Affinity constant
SDS-PAGE: SDS polyacrylamide gel electrophoresis
BmPPIase: *B. microti* peptidyl-prolyl cis-trans isomerase
IFA: Indirect immunofluorescence
GAGs: Glycosaminoglycan family
CSA: Chondroitin sulfate A
BMSA1: *B. microti* secret antigen 1
MSP1: Merozoite surface protein 1
HSP70: Heat shock protein 70

## Supporting Information

Supporting Information 1 Supporting Table 1: Parameters of Mass Spectrometry.

Supporting Information 2 Supporting Figure 1: *Babesia microti* before and after enrich. A for *B*. *microti* in erythrocytes, B for *B*. *microti* after enrich, and the photos were observed under a 10 ∗ 100x microscope.

Supporting Information 3 Supporting Figure 2: The SDS-PAGE analysis results of soluble proteins of *B. microti*. Analysis of these proteins by SDS-PAGE revealed five major bands with molecular weights of 72, 55, 43, and 17 kDa, along with seven secondary bands ranging from 14.4 to 95 kDa.

Supporting Information 4 Supporting Figure 3: The SDS-PAGE analysis of heparin affinity purification of *B. microti*. M represents marker, different lanes represent SDS-PAGE results of different components after affinity purification, Lane 1 represents natural proteins of *B. microti*, Lanes 2-3 represent flow-through fluid proteins of *B. microti*, Lanes 4-5 represent eluent of proteins of *B. microti*, and Lane 6 represents *B. microti* proteins bound to agarose.

Supporting Information 5 Supporting Figure 4: Expression and purification of peptidyl-prolyl cis-trans isomerase protein. (a) M represents marker, Lane A1 represents preinduced protein, Lane A2 represents induced protein, Lane A3 represents precipitation of induced protein, Lane A4 represents supernatant of induced protein. (b) M represents protein marker, B1 represents prepurified BmPPIase protein, B2 represents flow in liquid, Lanes B 3-7 represent purified BmPPIase protein in different concentration. (c) M represents protein marker, and Lane C1 represents 1 μl recombinant. BmPPIase and Lane C2 represents 2 μl recombinant. BmPPIase, lane C3 represents 1 μg BSA standard protein, Lane C4 represents 2 μg BSA standard protein, and Lane C5 represents 4 μg BSA standard protein. SA5 for recombinant BmSA5-1-1 protein.

Supporting Information 6 Supporting Figure 5: Cloning and expression of the BmSA5-1-1 protein. (a) M represents protein marker, Lane A1 represents preinduced protein, Lane A2 represents induced protein, Lane A3 represents supernatant of induced protein, and Lane A4 represents precipitation of induced protein. (b) M represents protein marker, Lane B1 represents prepurified protein, Lane B2 represents flow in liquid of protein, and Lanes B 3-7 represent purified protein in different concentration. (c) M represents protein marker, Lane C1 represents 1 μl recombinant BmSA5-1-1 protein, Lane C2 represents 2 μl recombinant BmSA5-1-1 protein, Lane C3 represents 1 μg BSA standard protein, Lane C4 represents 2 μg BSA standard protein, and Lane C5 represents 4 μg BSA standard protein.

## References

[1] Vannier E., Krause P. J. (2015). Babesiosis in China, an Emerging Threat. *The Lancet Infectious Diseases*.

[2] Chen M., Liu Q., Xue J. (2020). Spreading of Human Babesiosis in China: Current Epidemiological Status and Future Challenges. *China CDC Weekly*.

[3] Yao L. N., Ruan W., Zeng C. Y. (2012). Pathogen Identification and Clinical Diagnosis for One Case Infected with *Babesia*. *Zhongguo Ji Sheng Chong Xue Yu Ji Sheng Chong Bing Za Zhi*.

[4] Shih C. M., Liu L. P., Chung W. C., Ong S. J., Wang C. C. (1997). Human Babesiosis in Taiwan: Asymptomatic Infection with a *Babesia Microti-like* Organism in a Taiwanese Woman. *Journal of Clinical Microbiology*.

[5] Hsu N. H., Cross J. H. (1977). Serologic Survey for Human Babesiosis on Taiwan. *Taiwan Yi Xue Hui Za Zhi*.

[6] Zhou X., Li S. G., Chen S. B. (2013). Co-infections With *Babesia microti* and *Plasmodium* Parasites along the China-Myanmar Border. *Infectious Diseases of Poverty*.

[7] Sun Y., Li S. G., Jiang J. F. (2014). *Babesia venatorum* Infection in Child, China. *Emerging Infectious Diseases*.

[8] Jiang J. F., Zheng Y. C., Jiang R. R. (2015). Epidemiological, Clinical, and Laboratory Characteristics of 48 Cases of Babesia Venatorum Infection in China: A Descriptive Study. *The Lancet Infectious Diseases*.

[9] Man S. Q., Qiao K., Cui J., Feng M., Fu Y. F., Cheng X. J. (2016). A Case of Human Infection With a Novel Babesia Species in China. *IInfectious Diseases of Poverty*.

[10] Yokoyama N., Okamura M., Igarashi I. (2006). Erythrocyte Invasion by *Babesia* Parasites: Current Advances in the Elucidation of the Molecular Interactions between the Protozoan Ligands and Host Receptors in the Invasion Stage. *Veterinary Parasitology*.

[11] Chen Q., Barragan A., Fernandez V. (1998). Identification of *Plasmodium falciparum* Erythrocyte Membrane Protein 1 (PfEMP1) as the Rosetting Ligand of the Malaria Parasit*e P. Falciparum*. *Journal of Experimental Medicine*.

[12] Normark J., Nilsson D., Ribacke U. (2007). PfEMP1-DBL1*α* Amino Acid Motifs in Severedisease States of *Plasmodium falciparum* Malaria. *Proceedings of the National Academy of Sciences*.

[13] Zhang Y., Jiang N., Lu H. (2013). Proteomic Analysis of *Plasmodium falciparum* Schizonts Reveals Heparin-Binding Merozoite Proteins. *Journal of Proteome Research*.

[14] Bork S., Yokoyama N., Hashiba S. (2007). A Sexual Growth of *Babesia bovis* Is Inhibited by Specific Sulfated Glycoconjugates. *The Journal of Parasitology*.

[15] Suarez C. E., Florin-Christensen M., Hines S. A., Palmer G. H., Brown W. C., McElwain T. F. (2000). Characterization of Allelic Variation in the *Babesia bovis* Merozoite Surface Antigen1 (MSA-1) Locus and Identification of a Cross-Reactive Inhibition-Sensitive MSA-1 Epitope. *Infection and Immunity*.

[16] Tyler J. S., Treeck M., Boothroyd J. C. (2011). Focus on the Ringleader: The Role of AMA1 Inapicomplexan Invasion and Replication. *Trends in Parasitology*.

[17] Goo Y. K., Jia H., Aboge G. O. (2008). Babesia Gibsoni: Serodiagnosis of Infection in Dogs by an Enzyme-Linked Immunosorbent Assay with Recombinant BgTRAP. *Experimental Parasitology*.

[18] Sun J. H., Song P., Chen M. X. (2023). Expression and Functional Analysis of Recombinant Peptidyl⁃Prolylcis⁃Trans Isomerase Gene of *Babesia microti*. *Chinese Journal of Parasitology & Parasitic Diseases*.

[19] Cai Y. C., Wu F., Hu W. (2018). Molecular Characteriz Red Blood Cell Ation of *Babesia microti* Seroreactive Antigen 5-1-1 and Development of Rapid Detection Methods for Anti-*B. Microti* Antibodies in Serum. *Acta Tropica*.

[20] Thomas A. W., Narum D., Waters A. P. (1994). Aspects of Immunity for the AMA-1family of Molecules in Humans and Non-Human Primates Malarias. *Memorias do Instituto Oswaldo Cruz*.

[21] Mital J., Meissner M., Soldati D., Ward G. E. (2005). Conditional Expression of *Toxoplasma gondii* Apical Membrane Antigen-1 (TgAMA1) Demonstrates that TgAMA1 Playsa Critical Role in Host Cell Invasion. *Molecular Biology of the Cell*.

[22] Giovannini D., Späth S., Lacroix C. (2011). Independent Roles of Apical Membrane Antigen 1 and Rhoptry Neck Proteins during Host Cell Invasion by Apicomplexa. *Cell Host Microbe*.

[23] Boyle M. J., Richards J. S., Gilson P. R., Chai W., Beeson J. G. (2010). Interactions with Heparin-Like Molecules During Erythrocyte Invasion by *P. Falciparum* Merozoites. *Blood*.

[24] Chuang S. C., Yang C. D. (2014). Sustained Release of Recombinant Surface Antigen 2 (rSAG2) from Poly (Lactide-co-Glycolide) Microparticles Extends Protective Cell-Mediated Immunity Against *Toxoplasma gondii* in Mice. *Parasitology*.

[25] Terkawi M. A., Aboge G., Jia H. (2009). Molecular and Immunological Characterization of *Babesia gibsoni* and *Babesia microti* Heat Shock Protein-70. *Parasite Immunology*.

