# Supplementary material for: Proteomic Identification and Functional Analysis of Babesia microti Reveals Heparin-Binding Proteins
Source: J Trop Med. 2025 Jan 11;2025:8821002. doi: 10.1155/jotm/8821002 (PMC11742072; doi:10.1155/jotm/8821002)
Supplement: Supporting Information 1 — Supporting Table 1: Parameters of Mass Spectrometry. [file 8821002.f1.docx]

**Table 1. Parameters of Mass Spectrometry**

| **Items** | **Parameters** |
| --- | --- |
| database | Uniprot |
| classification | *Babesia microti* RI and PRA99（20759） |
| enzyme | trypsin |
| Dynamic modification | oxidize (M) |
| Fixed decoration | Aminomethyl (C) |
| Max Missed Cleavages | 2 |
| Peptide charge state | 1+，2+，3+ |
| Proteomic tools | 3.1.6 |
