# Supplementary figures and images for: Proteomic Identification and Functional Analysis of Babesia microti Reveals Heparin-Binding Proteins
Source: J Trop Med. 2025 Jan 11;2025:8821002. doi: 10.1155/jotm/8821002 (PMC11742072; doi:10.1155/jotm/8821002)

A

*Babesia microti*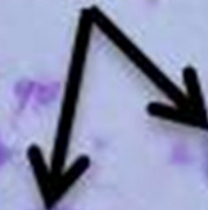

B

*Babesia microti*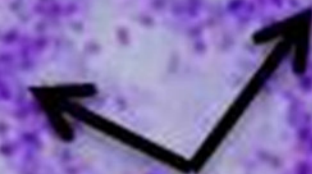

Supplement: Supporting Information 2 — Supporting Figure 1: Babesia microti before and after enrich. A for B. microti in erythrocytes, B for B. microti after enrich, and the photos were observed under a 10 ∗ 100x microscope. [file 8821002.f2.pdf]

**M**  
**180**  
**130**  
**95**  
**72**  
**55**  
**43**  
**34**  
**26**  
**17**  
**10**

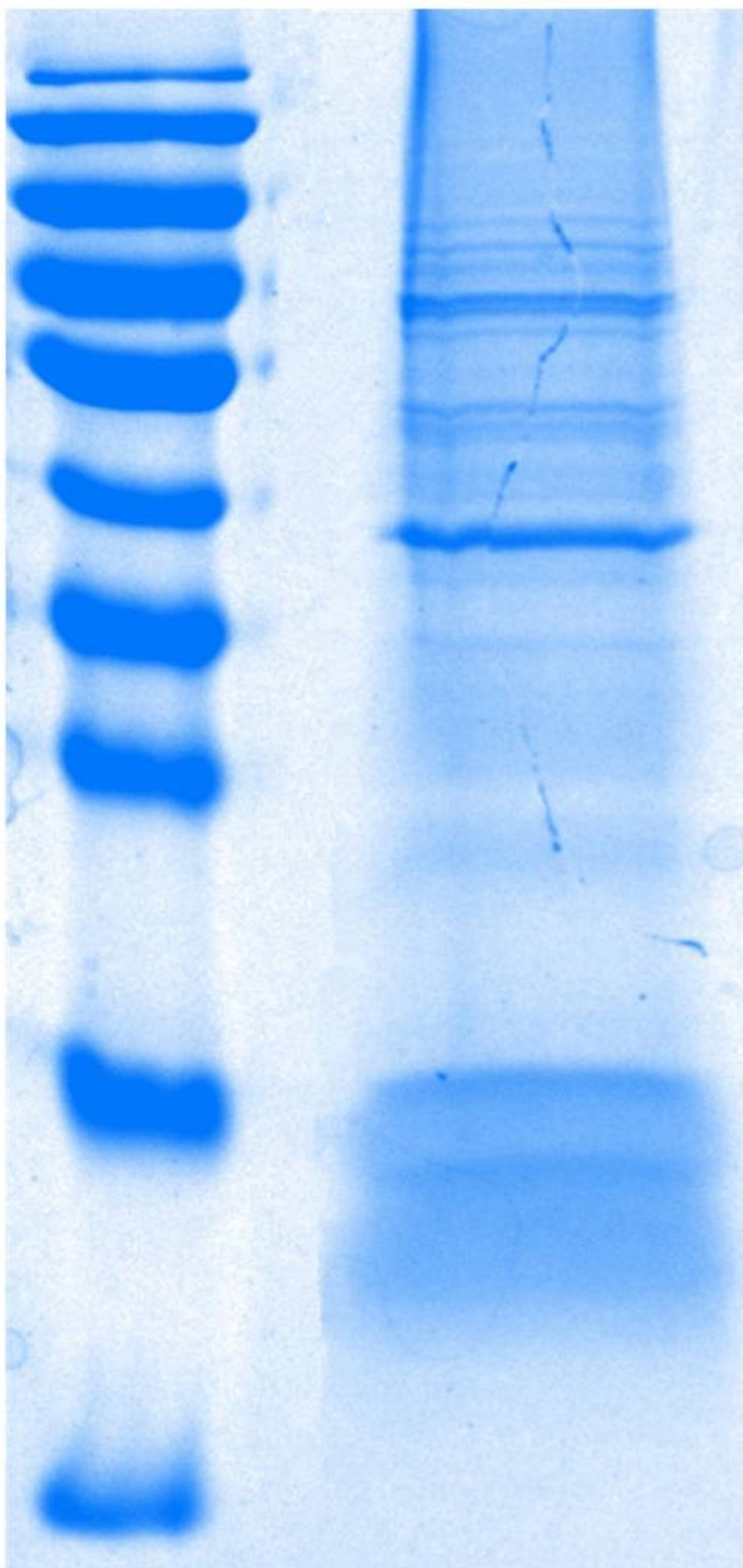

Supplement: Supporting Information 3 — Supporting Figure 2: The SDS-PAGE analysis results of soluble proteins of B. microti. Analysis of these proteins by SDS-PAGE revealed five major bands with molecular weights of 72, 55, 43, and 17 kDa, along with seven secondary bands ranging from 14.4 to 95 kDa. [file 8821002.f3.pdf]

M

1

2

3

4

5

6

180

130

95

72

55

43

34

26

17

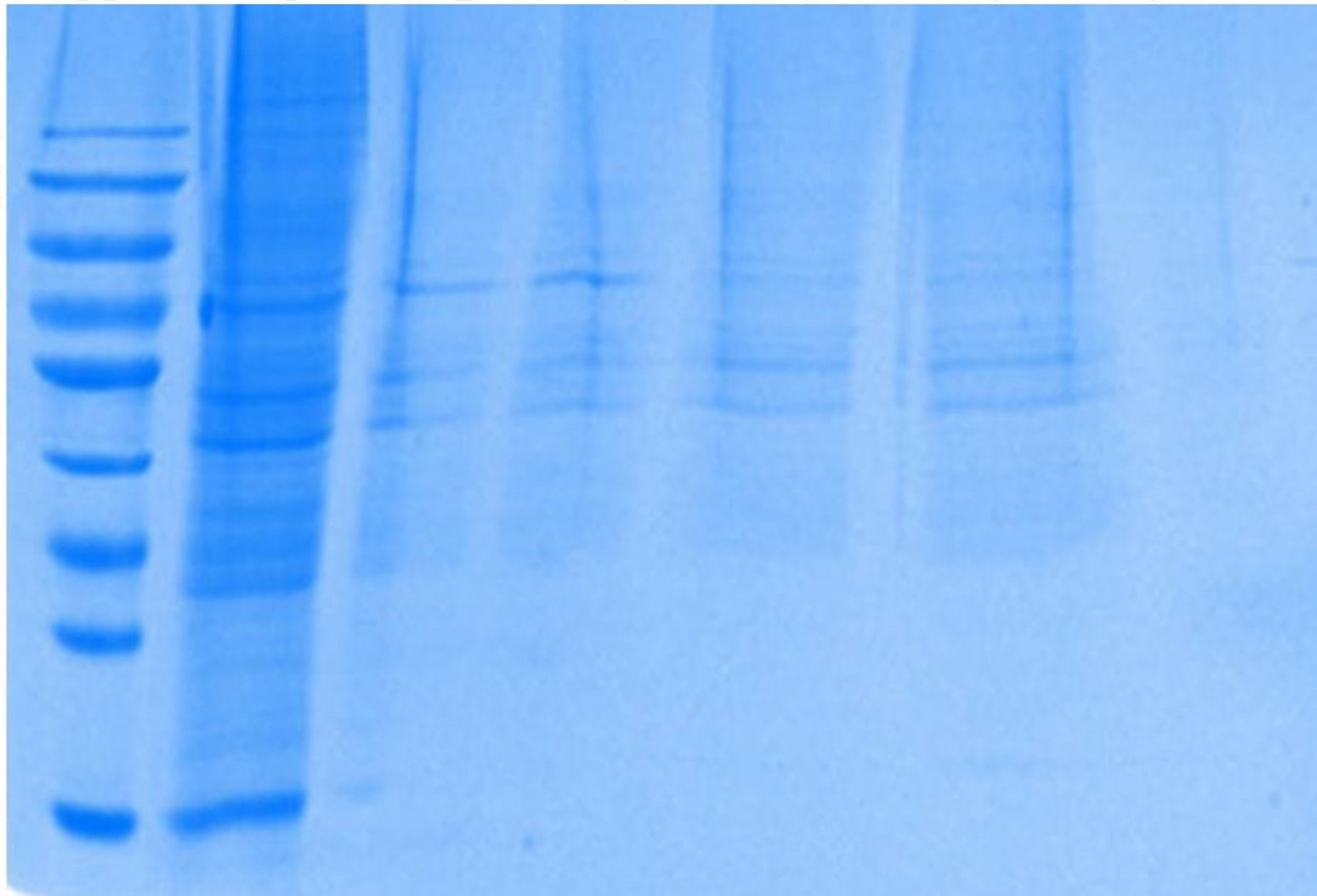

Supplement: Supporting Information 4 — Supporting Figure 3: The SDS-PAGE analysis of heparin affinity purification of B. microti. M represents marker, different lanes represent SDS-PAGE results of different components after affinity purification, Lane 1 represents natural proteins of B. microti, Lanes 2-3 represent flow-through fluid proteins of B. microti, Lanes 4-5 represent eluent of proteins of B. microti, and Lane 6 represents B. microti proteins bound to agarose. [file 8821002.f4.pdf]

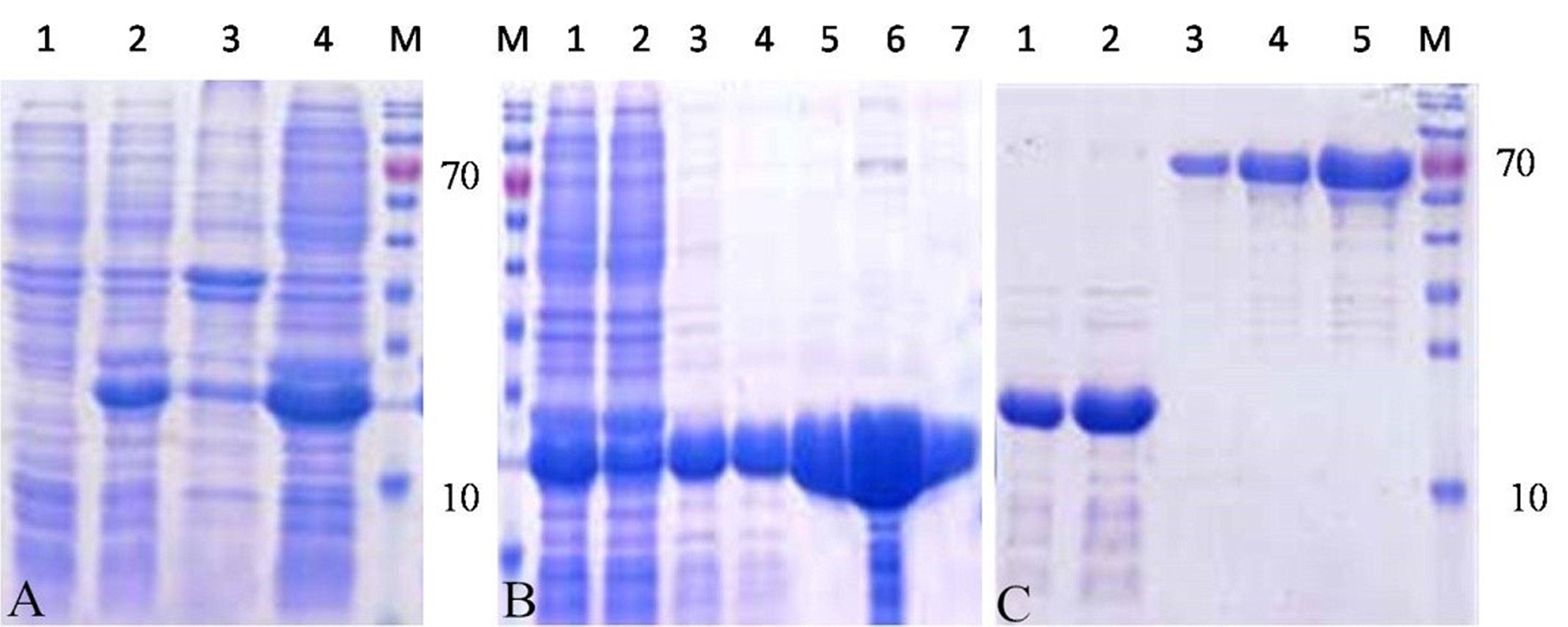

Supplement: Supporting Information 5 — Supporting Figure 4: Expression and purification of peptidyl-prolyl cis-trans isomerase protein. (a) M represents marker, Lane A1 represents preinduced protein, Lane A2 represents induced protein, Lane A3 represents precipitation of induced protein, Lane A4 represents supernatant of induced protein. (b) M represents protein marker, B1 represents prepurified BmPPIase protein, B2 represents flow in liquid, Lanes B 3-7 represent purified BmPPIase protein in different concentration. (c) M represents protein marker, and Lane C1 represents 1 μl recombinant. BmPPIase and Lane C2 represents 2 μl recombinant. BmPPIase, lane C3 represents 1 μg BSA standard protein, Lane C4 represents 2 μg BSA standard protein, and Lane C5 represents 4 μg BSA standard protein. SA5 for recombinant BmSA5-1-1 protein. [file 8821002.f5.pdf]

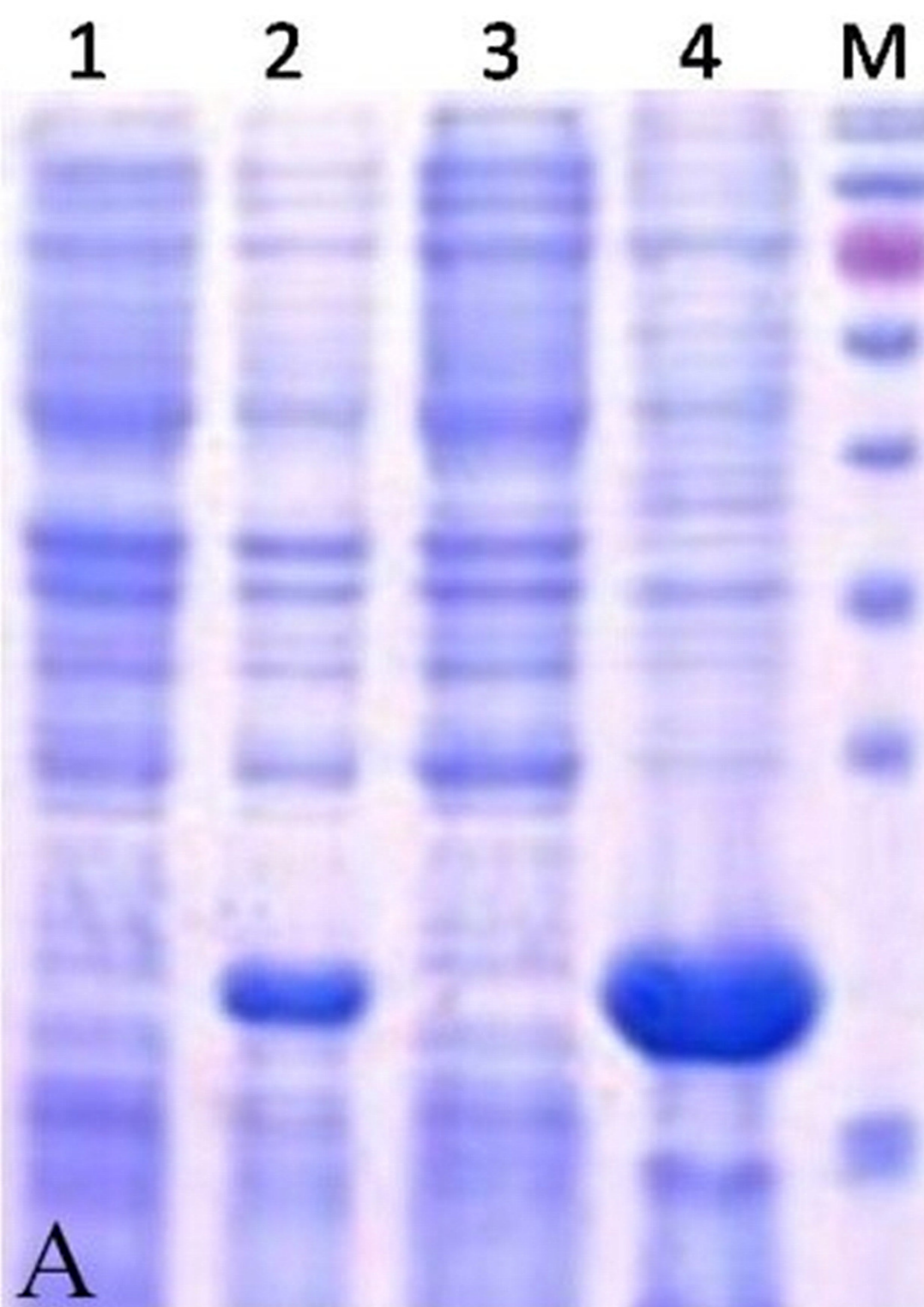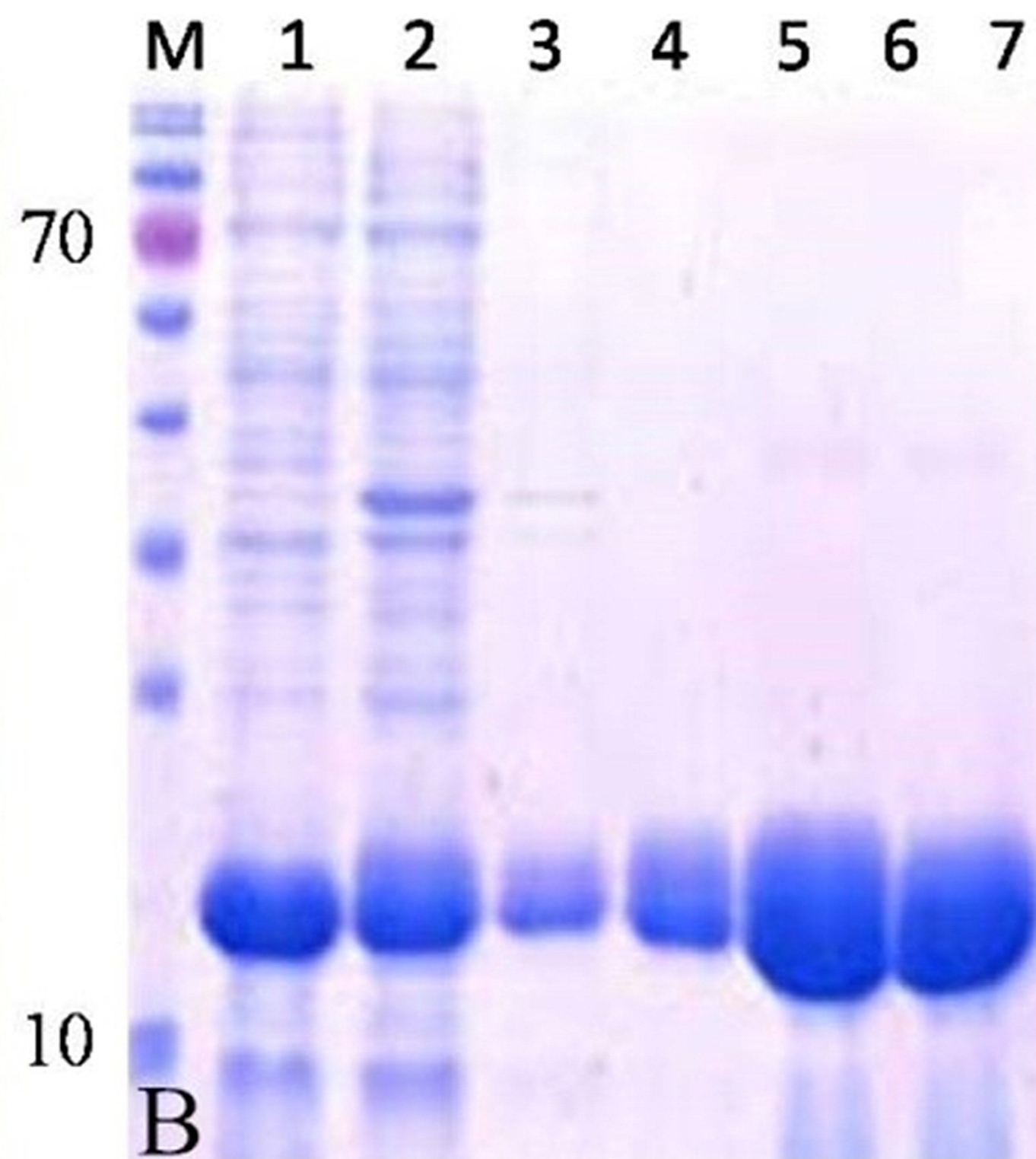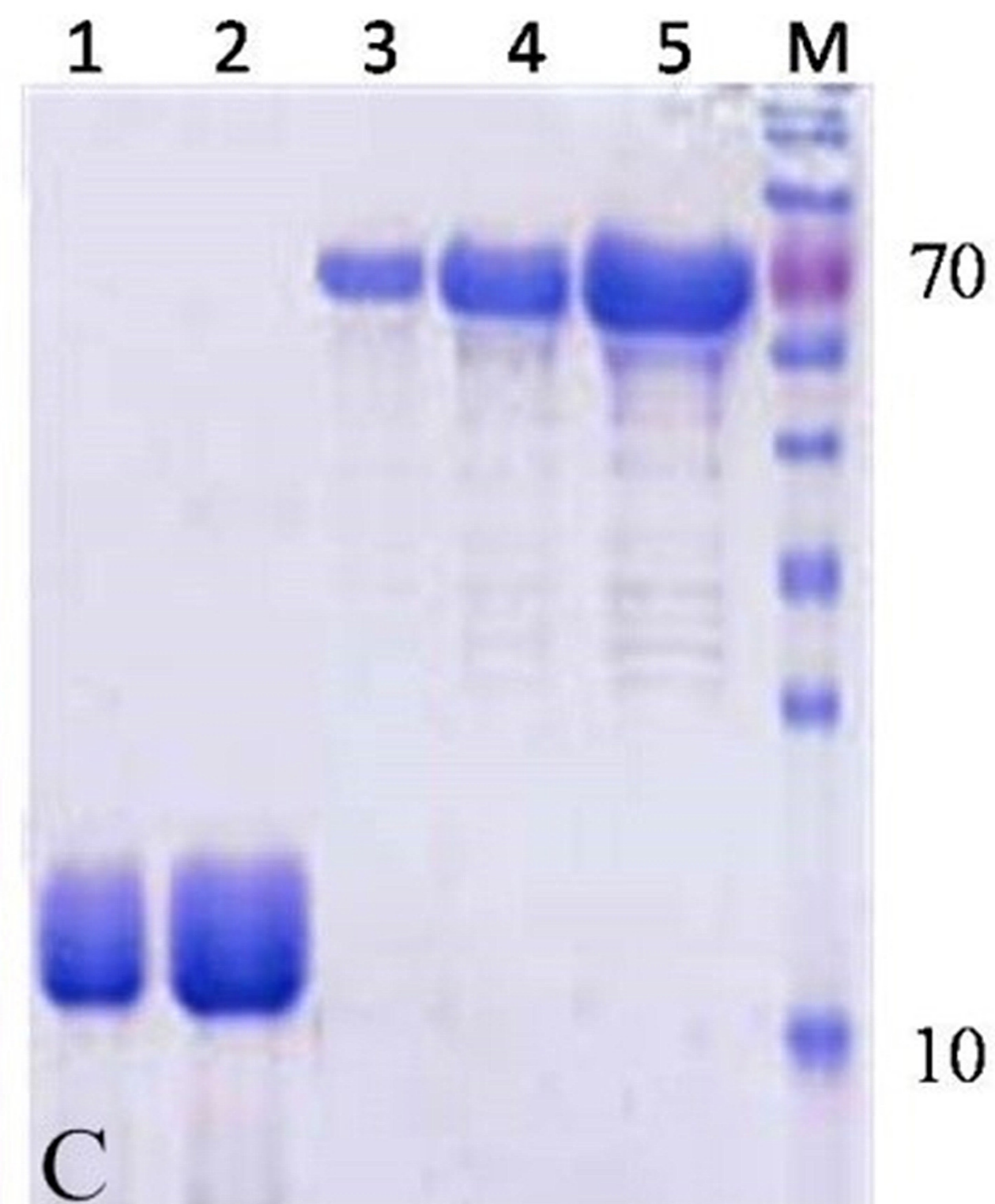

Supplement: Supporting Information 6 — Supporting Figure 5: Cloning and expression of the BmSA5-1-1 protein. (a) M represents protein marker, Lane A1 represents preinduced protein, Lane A2 represents induced protein, Lane A3 represents supernatant of induced protein, and Lane A4 represents precipitation of induced protein. (b) M represents protein marker, Lane B1 represents prepurified protein, Lane B2 represents flow in liquid of protein, and Lanes B 3-7 represent purified protein in different concentration. (c) M represents protein marker, Lane C1 represents 1 μl recombinant BmSA5-1-1 protein, Lane C2 represents 2 μl recombinant BmSA5-1-1 protein, Lane C3 represents 1 μg BSA standard protein, Lane C4 represents 2 μg BSA standard protein, and Lane C5 represents 4 μg BSA standard protein. [file 8821002.f6.pdf]
